# Genomic and phenotypic variation in epidemic-spanning *Salmonella enterica *serovar Enteritidis isolates

**DOI:** 10.1186/1471-2180-9-237

**Published:** 2009-11-18

**Authors:** Laura Betancor, Lucia Yim, Maria Fookes, Araci Martinez, Nicholas R Thomson, Alasdair Ivens, Sarah Peters, Clare Bryant, Gabriela Algorta, Samuel Kariuki, Felipe Schelotto, Duncan Maskell, Gordon Dougan, Jose A Chabalgoity

**Affiliations:** 1 Departamento de Desarrollo Biotecnológico, Instituto de Higiene, Facultad de Medicina, Universidad de la República, Av. A, Navarro 3051, CP 11600, Montevideo, Uruguay; 2 Departamento de Bacteriología y Virología, Instituto de Higiene, Facultad de Medicina, Universidad de la República, Av. A, Navarro 3051, CP 11600, Montevideo, Uruguay; 3 The Wellcome Trust Sanger Institute, Wellcome Trust Genome Campus, Hinxton, Cambridge CB10 1SA, UK; 4 Department of Veterinary Medicine, University of Cambridge, Madingley Road, Cambridge CB3 0ES, UK; 5 Centre for Microbiology Research, Kenya Medical Reserch Institute, Nairobi, Kenya

## Abstract

**Background:**

*Salmonella enterica *serovar Enteritidis (*S*. Enteritidis) has caused major epidemics of gastrointestinal infection in many different countries. In this study we investigate genome divergence and pathogenic potential in *S*. Enteritidis isolated before, during and after an epidemic in Uruguay.

**Results:**

266 *S*. Enteritidis isolates were genotyped using RAPD-PCR and a selection were subjected to PFGE analysis. From these, 29 isolates spanning different periods, genetic profiles and sources of isolation were assayed for their ability to infect human epithelial cells and subjected to comparative genomic hybridization using a *Salmonella *pan-array and the sequenced strain *S*. Enteritidis PT4 P125109 as reference. Six other isolates from distant countries were included as external comparators.

Two hundred and thirty three chromosomal genes as well as the virulence plasmid were found as variable among *S*. Enteritidis isolates. Ten out of the 16 chromosomal regions that varied between different isolates correspond to phage-like regions. The 2 oldest pre-epidemic isolates lack phage SE20 and harbour other phage encoded genes that are absent in the sequenced strain. Besides variation in prophage, we found variation in genes involved in metabolism and bacterial fitness. Five epidemic strains lack the complete *Salmonella *virulence plasmid. Significantly, strains with indistinguishable genetic patterns still showed major differences in their ability to infect epithelial cells, indicating that the approach used was insufficient to detect the genetic basis of this differential behaviour.

**Conclusion:**

The recent epidemic of *S*. Enteritidis infection in Uruguay has been driven by the introduction of closely related strains of phage type 4 lineage. Our results confirm previous reports demonstrating a high degree of genetic homogeneity among *S*. Enteritidis isolates. However, 10 of the regions of variability described here are for the first time reported as being variable in *S*. Enteritidis. In particular, the oldest pre-epidemic isolates carry phage-associated genetic regions not previously reported in *S*. Enteritidis. Overall, our results support the view that phages play a crucial role in the generation of genetic diversity in *S*. Enteritidis and that phage SE20 may be a key marker for the emergence of particular isolates capable of causing epidemics.

## Background

Infection with non-typhoidal *Salmonella enterica *is a major cause of food-borne disease in humans worldwide [1-3]. Animals and their products, particularly poultry and chicken eggs, are regarded as the main sources of this pathogen, although others, such as fresh vegetables, are also important [4-6]. A peculiar epidemiological feature of salmonellosis is that major outbreaks and epidemics are commonly associated with a dominant serovar of *S. enterica *and the particular serovar involved shows temporal and geographical variation.

Until the 1980s *S. enterica *serovar Typhimurium (*S*. Typhimurium) was the most common serovar isolated from humans worldwide. However, in the late 1980s *S*. Enteritidis emerged as the most common cause of human salmonellosis in Europe and during the 1990s it became the most prevalent serovar in many countries worldwide [7-9]. In Uruguay, until 1994 *S*. Typhimurium was the most frequently isolated serovar and *S*. Enteritidis was only isolated sporadically [10-12]. The first significant recorded outbreak of *S*. Enteritidis infection occurred in 1995 and from 1997 onwards it became the most prevalent serovar. After 2004 the number of isolates started to decline markedly, suggesting a post-epidemic period. The reasons for this worldwide serovar shift are still not understood, and several hypotheses have been proposed, including the existence of a rodent reservoir for *S*. Enteritidis, or the epidemiological change induced by vaccination of poultry against the closely related *S. enterica *serovar Gallinarum [13].

*S*. Enteritidis is highly clonal [14,15] so it has been difficult to discriminate genetic types by methods like multilocus sequence typing (MLST), pulsed field gel electrophoresis (PFGE), random amplified polymorphism DNA-PCR (RAPD-PCR) or ribotyping. DNA microarray-based comparative genomic hybridization (CGH) has been used to explore genetic diversity and to search for genes involved in virulence, transmission and host specificity in several different microbial pathogens [16-19] as well as in different serovars of *S. enterica *[20-26].

In this study we have genotyped 266 isolates of *S*. Enteritidis and defined a set of 29 isolates from before, during and after the epidemic period in Uruguay, covering different sources of isolation and representing the different profiles obtained by genotyping. To look for differences in pathogenic potential, these 29 isolates were assayed for their ability to invade Caco-2 epithelial cells. To correlate any differences in pathogenic potential with genomic variation we exploited a pan-*Salmonella *microarray for CGH. Six other *S*. Enteritidis isolated from distant parts of the world were included in the CGH analysis to compare the diversity seen in Uruguay with that found elsewhere.

## Results and Discussion

### Genotyping assays

All 266 *S*. Enteritidis isolates (Table 1) were subjected to RAPD-PCR analysis using 5 different primers and were compared to *S*. Enteritidis phage type 4 (PT4) strain P125109. The complete sequence of *S*. Enteritidis PT4 P125109 has been determined and it acts as the reference for all the analyses reported here [27].

**Table 1:** Uruguayan *S*. Enteritidis isolates included in this study.

	*ISOLATION PERIOD*			
**Sample origin**	**Pre-epidemic**	**epidemic**	**Post-epidemic**	**TOTAL**

Faeces	1	112	22	135
Blood	1	34	6	41
Urine	0	2	1	3
Spinal fluid	0	3	1	4
Other	0	9	2	11
**Subtotal human**	**2**	**160**	**32**	**194**
Food*	4	39	8	51
Animal	0	12	1	13
Feed	0	7	1	8
**Subtotal non-human**	**4**	**58**	**10**	**72**

**TOTAL**	**6**	**218**	**42**	**266**

*Includes eggs and other products used for human consumption.

Of the *S*. Enteritidis isolates tested in this study 96% showed the same amplification pattern as *S*. Enteritidis PT4 P125109 with all primers using RAPD-PCR. Only 10 isolates (3.8%) showed differences in the amplification pattern obtained with at least 1 primer.

Thirty-seven isolates from different origins, periods and RAPD types, were subjected to PFGE after cleavage of their DNA with *Xba*I. Of these, 26 generated a restriction pattern identical to *S*. Enteritidis PT4 P125109, whereas 11 showed subtle differences (1 to 3 different bands, corresponding to 96 to 91% identity with *S*. Enteritidis PT4 P125109). When both typing methods were considered together, 21 out of the 37 isolates were indistinguishable from *S*. Enteritidis PT4 P125109, while 5 differed by both methods and 11 differed by a single typing method. The 5 isolates differing by both methods included the 2 oldest pre-epidemic isolates (31/88 and 8/89), 2 isolated from food (206/99 and 32/02) and 1 isolated from human blood (214/02).

Overall these results revealed a high degree of genetic uniformity within *S*. Enteritidis circulating in Uruguay, with the great majority of isolates belonging to the same genetic profile as *S*. Enteritidis PT4 P125109.

Next, 29 isolates were selected with the aim of maximizing the chances of finding divergence among the isolates. For this, we selected isolates that span the pre-epidemic, epidemic and post-epidemic periods in Uruguay and that cover any particular profile found in the RAPD and/or PFGE assays, and all possible sources of isolation (Table 2). The selected isolates were subjected to further phenotypic and genotypic characterization.

**Table 2:** Description and results obtained for the Uruguayan isolates used for CGH and Caco-2 invasion assays

ISOLATE DESIGNATION	PHAGE TYPE	PERIOD OF ISOLATION	ORIGIN	RAPD-PCR^*a*^	PFGE^*b*^	CGH^*c *^P	CGH^*c *^A	SVP^*d*^	Caco-2 invasiveness^*e*^
31/88	UNTY	Pre epidemic	Coproculture	1	92%	32	43[4]	+	-
		
08/89	4b		Haemoculture	1	96%	33	41	+	-
		
53/94	4b		Food	0	100%	1	38	+	+
		
57/94	4		Food	0	100%	1	0[14]	+	+

47/95*	4	Epidemic	Coproculture	0	92%	2	0	+	+
		
51/95	4		Coproculture	1	100%	0	0	+	+
		
108/95*	4		Coproculture	0	100%	0	0	+	+
		
49/98	4		Food	0	100%	3	6[15]	+	+
		
80/98	4b		Bone punction	0	100%	0	1	+	+
		
100/99*	4		Coproculture	0	96%	0	0	+	+
		
130/99	4b		Coproculture	0	96%	0	0	-	-
		
132/99	4		Haemoculture	0	100%	0	0[33]	+	-
		
206/99	4b		Food	1	91%	0	45	-	+
		
32/00	4		Animal	0	100%	0	8[5]	+	+
		
125/00*	4		Coproculture	0	92%	0	0	+	+
		
48/01	4		Food	2	100%	0	0	+	-
		
251/01	RDNC		Egg	0	100%	0	0	+	-
		
254/01	4		Egg	0	96%	0	0	+	-
		
8/02*	4		Coproculture	0	100%	0	0	+	+
		
32/02	4		Egg	1	96%	0	1	-	+
		
65/02	4		Coproculture	0	100%	0	0	+	+
		
77/02*	4		Coproculture	0	100%	2	0	+	+
		
199/02	4		Haemoculture	1	ND	0	0	-	-
		
214/02	4		Haemoculture	1	96%	0	0	-	-
		
47/03	4		Coproculture	1	ND	0	6	+	+

106/04	4	Post epidemic	Coproculture	1	100%	0	0	+	+
		
10/05	RDNC		Coproculture	0	100%	2	0	+	+
		
92/05	4		Coproculture	0	96%	0	1[32]	+	+
		
93/05	4		Coproculture	0	100%	0	0	+	+

^***a***^- The number of primers which showed differences in RAPD -PCR profiles as compared to *S*. Enteritidis PT4 P125109 is indicated.

^*b*^- Expressed as percentage of identity as compared to *S*. Enteritidis PT4 P125109: 96% of identity corresponds to 1 band of difference, 92% to 2 bands and 91% to 3 bands of difference.

^*c*^- P: number of chromosomal genes predicted as present in test isolate but absent in reference *S*. Enteritidis PT4 P125109. A: number of chromosomal genes predicted as absent in test isolate but present in *S*. Enteritidis PT4 P125109. Numbers between brackets indicate genes from the *S*. Enteritidis PT4 P125109 virulence plasmid lost or divergent in the tested isolate (total plasmid genes: 57).

^*d*^- Results from Kado and Liu analysis [53]. +: *Salmonella *virulence plasmid present, -: absent.

^*e*^- Results from Caco-2 invasion assays. -: invasiveness ≤ 30% of PT4, +: > 30% of PT4. All isolates marked with - showed significant differences compared to PT4 (p < 0,01). In Isolate Designation column, an * indicates strains previously reported [12].

### Caco-2 invasion assays

In order to gain information about the pathogenic potential of *S*. Enteritidis, the 29 isolates plus the *S*. Enteritidis PT4 P125109 used as reference, were assayed for their ability to invade Caco-2 human epithelial cells (Table 2).

Contrary to the homogeneity observed when using the typing techniques, marked differences were observed between isolates in the cell invasion assays. Nine were impaired in their ability to invade (≤ 30% of the invasiveness of *S*. Enteritidis PT4 P125109; p < 0,01). These include the 2 oldest pre-epidemic isolates 31/88 and 8/89, 3 of 5 from human systemic disease, (132/99, 199/02 and 214/02), and 3 from food (48/01, 251/01 and 254/01). One particular isolate (130/99) defective in invasiveness was also impaired for growth in LB broth (data not shown). Of note, 7 out of these 9 isolates were distinct from *S*. Enteritidis PT4 P125109 when evaluated by RAPD or PFGE assays (see Table 2). All other isolates tested were similar to *S*. Enteritidis PT4 P125109 in this invasion assay. Considering all human isolates, 13 out of 15 obtained from gastroenteritis but only 1 out of 5 from invasive disease were as invasive as *S*. Enteritidis PT4 P125109 (p = 0,01 Fisher's exact test). Overall, these results suggest that impaired invasiveness is less frequent among isolates that cause human gastroenteritis, an assumption that merit future studies with a larger panel of *in vitro *and *in vivo *phenotypical assays.

### Comparative genomics of *S*. Enteritidis

These results suggest the existence of genetic determinants for the phenotypic differences that were not highlighted by the genotyping methods used. Consequently, we conducted a CGH study on the same 29 *S*. Enteritidis isolates from Uruguay used for the Caco-2 invasion assays. We also included in the CGH analysis 4 *S*. Enteritidis isolates from Kenya, and 2 isolates from the UK as external comparators.

The analysis was conducted using a pan-*Salmonella *microarray based on the *S*. Typhi CT18 genome, complemented with strain-specific genes from *S*. Enteritidis PT4 P125109, *S*. Typhimurium SL1344 and DT104, *S*. Gallinarum, *S*. Typhi Ty2 and *S. bongori *(see methods). Genes specific for some of these strains were not included in previously reported *S*. Enteritidis CGH analysis. Of 5863 features on the microarray, 3978 correspond to genes present in *S*. Enteritidis PT4 P125109 (3921 chromosomal and 57 plasmid genes) and 1885 to genes absent in *S*. Enteritidis PT4 P125109 but present in other salmonellae.

Overall, the analysis produced results that extend those previously reported by others using different sets of isolates [21,24,25], and confirm that there is considerable genetic homogeneity in *S*. Enteritidis, despite geographical, temporal and source differences between the different isolates. However, we also found a number of genomic regions and single genes that have not been described as variable among *S*. Enteritidis field isolates.

Of the 3921 chromosomal genes from *S*. Enteritidis PT4 P125109 represented on the microarray (covering about 90% of the genome), 3804 were shared by all *S*. Enteritidis isolates tested here and are considered to be the core genome of *S*. Enteritidis. Among these genes, only 7 were specific to *S*. Enteritidis, i.e. absent in all other sequenced *Salmonella *strains, and they are all included in the recently annotated phage SE14 [27]. Interestingly, this region was previously postulated as a region of difference between *S*. Enteritidis and other serovars [28], although more recently it was reported as absent in two *S*. Enteritidis isolates corresponding to PT6b and PT35 (Region A04 in reference [21]).

**Figure 1:**
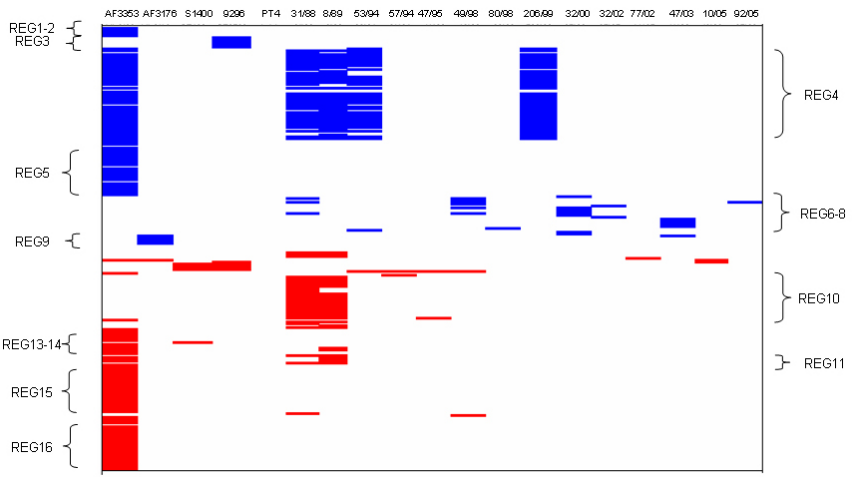
**Graphic representation of the chromosomal genes found in this study as part of *S*. Enteritidis Dispensable Genome (233 genes)**. In blue, genes present in the *S*. Enteritidis PT4 P125109 chromosome and predicted as absent in the test strain. In red, genes absent in the *S*. Enteritidis PT4 P125109 chromosome and predicted as present in the test strain. In white, genes present or absent in both reference and test strains. Only those isolates for which any divergence is predicted are shown. *S*. Enteritidis PT4 P125109 results are shown as reference.

Considering genes that were variably present between the isolates tested, 117 genes known to be present on the chromosome of *S*. Enteritidis PT4 P125109 were absent, or divergent, in at least one of the *S*. Enteritidis isolates tested (Regions 1 to 9 and single genes 1 to 9, see Table 3). Conversely 116 genes were present in at least one isolate but absent from *S*. Enteritidis PT4 P125109 (Regions 10 to 16 and individual genes 10 to 26, see Table 4). These results are summarized in Figure 1. These 233 genes together with other 201 genes previously described as variable present in *S*. Enteritidis [21] can be considered so far the *S*. Enteritidis dispensable genome (DG). Of note, 10 of the 16 regions of variability (Reg 1, 3, 5-8, 10, 12-14) are reported for the first time as being variable among *S*. Enteritidis strains. Variation in plasmid genes is not included in this figure and has been treated separately (see below).

**Table 3:** Regions (REG) and single genes (SING) present in the *S*. Enteritidis PT4 P125109 chromosome and predicted by CGH analysis as absent or divergent in at least one *S*. Enteritidis isolate.

	ISOLATE DESIGNATION	GENE RANGE	HOMOLOGOUS^a^	FUNCTION/GENE PREDICTION
**REG 1**	AF3353	SEN0910-SEN0912	No	Part of SE10 prophage remnant.

**REG 2#**	AF3353	SEN1394-SEN1395	No	Part of SE14 prophage.

**REG 3**	9296/98	SEN1524-SEN1530	CT18, TY2, LT2, DT104, SL1344, SBG, SPA, SGAL	Membrane, transport and hypothetical proteins

**REG 4#**	31/88, 8/89, 53/94, 206/99, AF3353	SEN1920-SEN1966	SDT104	Phage SE20

**REG 5**	AF3353	SEN1970-SEN1999	SGAL	Genomic island ROD 21, coding to HNS

**REG 6**	31/88, 49/98, 92/05 (onlySEN2240)	SEN2238-SEN2243	CT18, TY2, LT2, DT104, SL1344, SBG, SPA, SGAL	CytochromeC synthesis, ferredoxin

**REG 7**	32/00, 31/88, 49/98	SEN2441-SEN2446	CT18, TY2, LT2, DT104, SL1344, SPA, SGAL	Alcohol dehydrogenase, aldehyde dehydrogenase, ethanolamine utilization

**REG 8**	47/03	SEN2761-SEN2763	CT18, TY2, LT2, DT104, SL1344, SBG, SPA, SGAL	*rpoS*, unknown

**REG 9#**	AF3176, 47/03 (only SEN4286)	SEN4286-SEN4291	SGAL	ROD40, Type I Restriction Modification System Methyltransferase

**SING 1**	32/00	SEN2051	CT18, TY2, LT2, DT104, SL1344, SPA, SGAL	*pduS *(ferredoxin)

**SING 2**	32/02	SEN2293	CT18, TY2, LT2, SL1344, SBG, SPA, SGAL	Hypothetical Protein

**SING 3**	32/00	SEN2494	CT18, LT2, SL1344, DT104, SGAL	*ratB*, lipoprotein

**SING 4**	47/03	SEN2819A	CT18, TY2, DT104, SL1344, SPA, SGAL	*fucP*, L-fucose permease

**SING 5**	47/03	SEN2841	CT18, TY2, LT2, DT104, SL1344, SBG, SPA, SGAL	*ppdB*, prepilin peptidase dependent protein B precursor

**SING 6**	80/98	SEN2912	CT18, TY2, LT2, DT104, SL1344, SBG, SPA, SGAL	*pgk*, phosphoglycerate kinase

**SING 7**	53/94	SEN3403	CT18, TY2, LT2, DT104, SL1344, SBG, SPA, SGAL	Lipoprotein

**SING 8**	32/00	SEN3627	CT18, TY2, LT2, DT104, SL1344, SBG, SPA, SGAL	*yidR*, putative ATP/GTP binding protein

**SING 9**	32/00	SEN3636	CT18, TY2, LT2, DT104, SL1344, SBG, SPA, SGAL	*yhjA*, probable cytochromeC peroxidase

^a ^indicates when the REG or SING has homologous region described in other sequenced *Salmonella *serovars (see list of abbreviations). **# **indicates that the genetic region was previously described as variable among *S*. Enteritidis isolates [21].

**Table 4:** Regions (REG) and single genes (SING) absent in the *S*. Enteritidis PT4 P125109 chromosome and predicted by CGH analysis as present in at least one Enteritidis isolate.

	ISOLATE DESIGNATION	GENE RANGE	HOMOLOGOUS^a^	GENE DESCRIPTION
**REG 10A**	31/88	SDT1842-SDT1843	No	Similar to E coli K12 *ymfD, ymfE *phage proteins

**REG 10B**	31/88, 8/89, 47/95 (only SDT1860)	SDT1846-SDT1860	No	Shigella Phage proteins

**REG 11#**	8/89, AF3353, 31/88 (only STY1036)	STY1034-STY1036	SL1344, LT2, TY2, DT104	Part of Gifsy-2 antitermination *ninG, dnaJ*

**REG 12A**	31/88, 8/89	SL2583-SL2584	SBG	Phage related protein

**REG 12B**	31/88, 8/89	SL2588-SL2594	some SBG	Phage proteins, putative methyltransferase, unknown

**REG 12C**	31/88, 8/89	SL2599-SL2600	LT2, SDT104	Gifsy-1 integrase, unknown

**REG 13**	AF3353, 8/89 (only STY1013)	STY1011-STY1013	TY2, LT2, SL1344, DT104	Phage proteins (integrase, excisionase)

**REG 14**	AF3353, 8/89 (only STY1021)	STY1021-STY1024	TY2, LT2, SL1344, DT104	Phage proteins

**REG 15A#**	AF3353	STY3674-STY3689	SL1344, LT2, TY2, SPA	ST35 phage proteins

**REG 15B**	AF3353	STY3696-STY3702	TY2, SPA, LT2, SL1344	ST35 phage proteins

**REG16A**	AF3353	STY4600-STY4602	TY2, SPA. LT2, SL1344, SBG (except 4600)	Part of S. Typhi phage SopE

**REG16B**	AF3353	STY4605-STY4607	TY2, SPA, LT2, SL1344, SBG	Part of S. Typhi phage SopE

**REG16C#**	AF3353	STY4613-STY4628	TY2, SPA. LT2, SL1344 (except 4619)	Part of S. Typhi phage SopE

**REG16D#**	AF3353	STY4633-STY4635	SL1344, LT2, SPA	Part of S. Typhi phage SopE

**REG16E**	AF3353	STY4638-STY4639	TY2, SPA, LT2. SL1344 (except 4639)	Part of S. Typhi phage SopE

**REG16F**	AF3353	STY4641-STY4645	TY2, SPA. LT2 (except 4641)	Part of S. Typhi phage SopE

**SING 10**	53/94, 57/94, 47/95, 49/98	SBG0310	No	unknown

**SING 11**	31/88	SBG3602	LT2, CT18	Hypothetical protein

**SING 12**	S1400/94	STY0114	TY2, SPA	Putative IS transposase

**SING 13**	77/02	STY0480	TY2, SPA	Hypothetical protein

**SING 14**	49/98	STY4582	No	Exported protein

**SING 15**	31/88	STM0293	SL1344, DT104	unknown

**SING 16**	31/88	SDT2674	SL1344	unknown

**SING 17**	31/88, 8/89	STM2584	DT104, SL1344	*gogB*, leucine-rich repeat protein

**SING 18**	49/98	STY3619	TY2, SPA, LT2, SL1344	Conserved membrane protein

**SING 19**	AF3353	SBG0897	SBG	Phage related protein

**SING 20**	AF3353	SDT1865	No	unknown

**SING 21**	AF3353	SDT3861	No	unknown

**SING 22**	AF3353	STY1073	LT2, TY2	unknown

**SING 23**	AF3353	STY2013	TY2	unknown

**SING 24**	AF3353	STY4600	TY2, SPA	Transcriptional regulator

**SING 25**	AF3353	STY4619	TY2, SPA	Putative membrane protein

**SING 26**	AF3353	STY4639	TY2, LT2, SPA	Hypothetical protein

^a ^indicates when the REG or SING has homologous region described in other sequenced *Salmonella *serovars (see list of abbreviations). **# **indicates that the genetic region was previously described as variable among S. Enteritidis isolates [21].

Detailed analysis of the genes within the DG showed that prophage-like elements constitute the major source of genetic variation distinguishing these *S*. Enteritidis isolates. However, this analysis also revealed some interesting differences in metabolic potential and in genes associated with restriction-modification systems (discussed below).

#### *S*. Enteritidis variable prophage-like regions within the DG

Of the annotated prophages from *S*. Enteritidis PT4 P125109 represented on the array one Kenyan and 4 Uruguayan isolates lacked ϕSE20 (Region 4 in our analysis), a ~41 kb phage similar to ϕST64B. Phage SE20 is thought to be intact and a recent acquisition in *S*. Enteritidis PT4 P125109 and like ϕST64B, it carries fragments of the *sopE *and *orgA *genes, which have been implicated in *Salmonella *virulence [27,29]. Two of the 4 Uruguayan isolates that lack ϕSE20 were isolated from human infections more than 5 years before the beginning of the epidemic in Uruguay (31/88 and 8/89), whereas the other 2 were from food samples, one from before (53/94) and the other from the middle (206/99) of the epidemic. Similarly, Porwollik and collaborators have reported that this phage (called ϕST64B in their work) is absent in strains of *S*. Enteritidis isolated more than 50 years ago and suggested that acquisition of this phage may be related to the emergence of *S*. Enteritidis as being epidemic worldwide [21].

We corroborated the presence of ϕSE20 among the 29 Uruguayan isolates by PCR using two set of ϕSE20-specific primers that amplify fragments of *sb9 *and *sb41 *(SEN1935 and SEN1993 respectively). Only isolates 31/88, 8/89, 53/94 and 206/99 were negative validating the microarray results. We extended the PCR screening with *sb41 *primers to another 85 *S*. Enteritidis isolates from the original sample set, which included 28 isolates from human gastroenteritis, 30 isolates from invasive human disease and 27 isolates from non-human origin (including the 2 other pre-epidemic isolates that had not been included in the CGH analysis). Among them we found only 4 other isolates that lack *sb41*, i.e. 50/99 and 211/00 originating from food, 107/99 from enteric disease and 209/01 from invasive infection. In summary, we found that only 5 out of 108 isolates tested from the epidemic and post-epidemic periods lack ϕSE20, whereas 3 out of 6 pre-epidemic isolates lack this phage. This provides further support for the idea that the presence of ϕSE20 is a marker for the emergence of particular isolates as epidemic strains [21,27].

It has been proposed that *S*. Enteritidis might be divided into two lineages based on the presence or absence of four phages, i.e. ϕSE20, Fels2 and *S*. Typhi CT18 ST27 and ST35 phages [21]. One lineage, the PT4 lineage, was defined as positive for ϕSE20 and negative for Fels2, ST27 and ST35, whereas a second lineage, the PT8-PT13 lineage, was defined as negative for ϕSE20 but positive for Fels2, ST27 and ST35. Our results however, show that all Uruguayan isolates tested belong to the PT4 lineage as defined by Guard-Petter [30], and are negative for Fels2, ST27 and ST35 phage regions regardless of the presence or absence of ϕSE20, thus they do not strictly fall within the two separates groups as previously proposed [21].

Several prophage-related genes present on the microarray from other non-*S*. Enteritidis serovars were found in some of the isolates. Many of them are grouped here as regions 10 to 16 (Table 4). Regions 15 and 16 were only found in the Kenyan *S*. Enteritidis AF3353 isolate. Region 15 encodes 23 (out of 45) genes corresponding to sequences of the *S*. Typhi CT18 P2-family prophage ST35 [31]. Region 16 harbours 32 genes from another P2-family prophage, ϕSopE, also found in *S*. Typhimurium and *S*. Typhi that encodes the type III secretion system effector protein SopE important for invasion of enterocytes [31-33]. In *S*. Enteritidis, SopE is encoded in an unrelated lambdoid phage SE12 [27,33], which is present in all *S*. Enteritidis isolates tested here.

We found that the two oldest Uruguayan pre-epidemic isolates (31/88, 08/89) harbour 31 genes (regions 10 to 12) that correspond to phage genes carried by *S*. Typhimurium DT104 or *S*. Typhimurium SL1344, or genes from ϕGifsy-1 of *S*. Typhimurium LT2. Interestingly, Regions 10 and 12A-B were not previously found in *S*. Enteritidis, although this may be due to the fact that previously reported *S*. Enteritidis CGH analysis used microarrays that lacked these regions.

Both pre-epidemic isolates also carry *gogB*. GogB is a ϕGifsy-1-encoded type III secreted substrate of both SPI-1 and SPI-2 TTSS in *S*. Typhimurium LT2 [34]. It has been reported that some salmonellae have Gifsy-1 but not *gog*B whereas others do not have Gifsy-1 but do have *gogB*, suggesting that this gene has been recently acquired by Gifsy-1 [34,35]. To the best of our knowledge, this is the first report of *S*. Enteritidis harbouring this gene. Thus, we designed a pair of primers that amplifies a 248 bp fragment of *gogB*, and used them to screen for its presence among the 85 strains also assayed for ϕSE20. No other isolate was positive for *gogB*. We then sequenced the PCR fragment from both pre-epidemic strains and found that the sequence has 99% of identity with *S*. Typhimurium LT2 *gogB*.

In summary, 10 out of the 16 variable genomic regions found among *S*. Enteritidis isolates correspond to phage-like regions, suggesting that, as in other serovars of *Salmonella*, phages play a crucial role in the generation of genetic diversity in *S*. Enteritidis [20,31].

#### Variations in the content of genes involved in metabolism

Our CGH data highlighted other regions of variation. Region 7, harbouring 6 out of 17 genes of the *eut *operon, is absent in 1 pre-epidemic (31/88) and 2 non-human epidemic (32/00 and 49/98) *S*. Enteritidis isolates. These genes encode alcohol dehydrogenase, aldehyde dehydrogenase and enzymes required for ethanolamine utilization (*eutG, J, E, N, M, D*). *S*. Enteritidis 32/00 also lacks the *pduS *gene, a ferredoxin involved in propanediol utilization (part of the *pdu *operon). In *Salmonella *both 1, 2-propanediol degradation and ethanolamine degradation require vitamin B_12_. Many *Enterobacteriaceae *have lost the capacity to synthesize cobalamine and therefore to degrade 1, 2-propanediol and ethanolamine but a few genera, including *Salmonella *and *Yersinia*, re-acquired a 40 kb metabolic island encoding both the ability to synthesise cobalamine and degrade 1, 2-propanediol, whilst retaining the *eut *operon [36-39]. Although 1, 2-propanediol is an important source of energy for *S*. Typhimurium and *cbi *mutants are significantly attenuated in their ability to grow in macrophages [40] it is apparent that genes within these pathways are lost in the host-adapted *S. enterica *serovars including Gallinarum, Typhi and Paratyphi A [27].

Region 8 (SEN2761-SEN2763) comprises three genes (*rpoS *and two unknown genes) which are absent/divergent in *S*. Enteritidis 47/03 isolated from human disease. RpoS is inducible in stationary phase, is the master regulator of the general stress response in *Salmonella *and is required for virulence in mice [41,42]. There are previous reports of *S*. Typhi, *S*. Typhimurium and *S*. Enteritidis clinical and environmental isolates carrying mutations in *rpoS *that result in impaired RpoS functionality [42,43]. A test of catalase activity in stationary phase is used as a method to detect RpoS function [42], thus we performed the test in all 29 isolates and found a negative result only in *S*. Enteritidis isolate 47/03. This strongly suggest that RpoS function is impaired in this isolate.

Region 6 harbouring genes encoding nitrate reductases, cytochrome C and ferredoxin-type proteins (*napC, B, H, G, A, D*), was also absent in 3 *S*. Enteritidis (31/88, 48/98 and 92/05) isolates from different periods of the Uruguayan epidemic.

#### Variation in *S*. Enteritidis Genomic Islands

Although there is a large number of genomic islands in *S*. Enteritidis PT4 P125109 [27] which carry the hallmarks of having been laterally acquired, and maintain mobility functions, surprisingly our data show that most are ubiquitous in the *S*. Enteritidis isolates tested here. The exceptions are Region 5 (or ROD21) and Region 9. Region 5 is one of the largest genomic islands identified in *S*. Enteritidis PT4 P125109 (26.5 kb; SEN1970-SEN1999), and it encodes the global transcriptional silencers H-NS (*hnsB*) and the H-NS antagonist (*hnsT*) [44-46]. This region was undetected using the microarray in the Kenyan *S*. Enteritidis isolate AF3353 but it is present in all other strains. Region 9 corresponds to the immigration control region ICR in *S*. Enteritidis PT4 P125109 [27] which encodes two type I restriction/modification systems. All of these genes were not detected in the Kenyan *S*. Enteritidis isolate AF3176 and partially detected in isolate 47/03, which lacks one of the restriction enzyme subunits.

In addition to variation in genes found in large clusters in *S*. Enteritidis PT4 P125109 there was also variation in genes found as singletons (summarised in Tables 3 and 4). Of note is the absence of the gene *ratB *in *S*. Enteritidis isolate 32/00. This gene is located within the CS54 genomic island in *S*. Typhimurium, a region that is important for intestinal persistence in a mouse model [47]. In *S*. Enteritidis PT4 P125109, the genomic island is maintained but *ratB *is a pseudogene, as it is in the sequenced strains of the host-adapted serovars *S*. Typhi and *S*. Gallinarum.

#### Variation in plasmid-encoded genes

**Figure 2:**
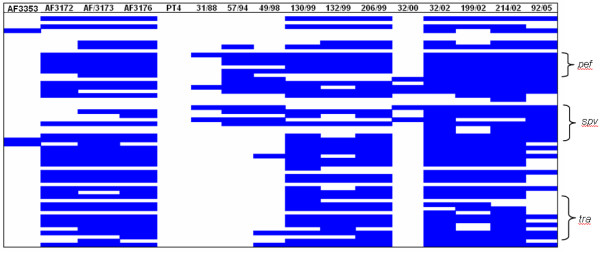
**Graphical representation of the 57 genes from the *Salmonella *virulence plasmid as found in isolates that showed differences in plasmid content by CGH**. In blue, genes present in the *S*. Enteritidis PT4 P125109 virulence plasmid and predicted as absent in the test strain. In white, genes present in both reference and test strains.

Besides chromosomal genes, the microarray incorporated genes found on *Salmonella *virulence plasmids from serovars Enteritidis, Gallinarum, Typhimurium and plasmids, pHCM1 and pHCM2, from the multi-drug resistant *S*. Typhi strain CT18. Five Uruguayan isolates, 2 from food (206/99 and 32/02) and 3 from human disease (130/99, 199/02 and 214/02), lack the characteristic *S*. Enteritidis virulence plasmid. This was confirmed by attempts to purify the plasmid (Table 2). Two other Uruguayan isolates, 92/05 and 132/99, exhibited divergence in more than 30 genes and isolates 57/94 and 49/98 diverged in 15 genes found within the plasmid of *S*. Enteritidis PT4 P125109 (see Table 2 and Figure 2). Included in the genes predicted as absent or divergent are the *spv *genes, the *pef *fimbrial operon as well as *repA *(DNA replication) and *rsdB *(resolvase). Of note, isolates 92/05 and 132/99 also lack the few *tra *genes remaining in *S*. Enteritidis PT4 P125109.

Despite the high degree of variability seen in these plasmids all had similar molecular weights when compared to that in *S*. Enteritidis PT4 P125109 (data not shown), suggesting potential divergence in gene sequence or acquisition of novel genes. However none of the isolates with high variation in plasmid gene content showed a positive signal for non-*S*. Enteritidis plasmid features included in the array, suggesting that they may harbour sequence divergence or novel sequences. In fact the only isolate showing a positive signal for non-*S*. Enteritidis plasmid features was the Kenyan *S*. Enteritidis isolate AF3353 which harbours the complete *S*. Enteritidis PT4 P125109 virulence plasmid and shows a positive signal for 10 genes from the multidrug resistance plasmid, pHCM1, from *S*. Typhi CT18. These include genes encoding β-lactamase and streptomycin resistance. Although we cannot confirm that these are located on the plasmid there are increasing numbers of reports of drug resistance genes integrating into the virulence plasmid [48,49].

## Conclusion

The results presented here corroborate and extend previous reports demonstrating a high degree of genetic homogeneity among field isolates of *S*. Enteritidis, irrespective of geographical, temporal and source differences. Most of the strains analysed produced highly similar profiles by RAPD and PFGE analysis, and those selected for further analysis showed almost indistinguishable gene content by microarray-based CGH. The two oldest Uruguayan pre-epidemic *S*. Enteritidis isolates and a Kenyan isolate (AF3353) were among the most divergent. Most of the genome variation was related to prophage regions underscoring their importance as drivers for *S*. Enteritidis evolution. In particular half of the isolates from before the beginning of the *S*. Enteritidis epidemic in Uruguay lack ϕSE20, whereas absence of this phage is minimal (less than 5%) among *S*. Enteritidis isolated during and after the epidemics, as detected by CGH and extended by PCR screening. These results, together with those previously reported [21] strongly suggest that this phage may have been relatively recently acquired by *S*. Enteritidis, and that this might be related to the capacity of PT4-like strains to become prevalent. Although we are aware that the small number of pre-epidemic isolates is a limitation of this study, it is noteworthy that these are all the *S*. Enteritidis isolates received at the National Salmonella Centre since the beginning of the 1970s until the end of 1994.

The two oldest pre-epidemic isolates also carry genetic regions that were not found in *S*. Enteritidis strains previously evaluated by CGH [21,24,25], but this may be due to the fact that more genes from other serovars of *Salmonella *are present on our microarray compared with those previously reported. Beside these, we have confirmed that 2 Uruguayan isolates harbour *gogB*, a gene that has not been previously found among *S*. Enteritidis strains.

In addition to identifying differences in the content of mobile genetic elements we were successful in identifying metabolic pathways which appear to be incomplete in some isolates. These include those associated with the utilization of propanediol and ethanolamine as well as many genes that have previously been implicated in bacterial fitness and virulence (e.g. global transcriptional silencers H-NS, immigration control region ICR, *rpo*S, *gogB*, *ratB*). We also showed that a significant number of the Uruguayan *S*. Enteritidis strains lack the *Salmonella *virulence plasmid and others showed variation in plasmid gene content.

There was great heterogeneity in the ability of the isolates to invade Caco-2 human epithelial cells, but our genotyping approach was insufficient to elucidate the genetic bases for these differences. These finding are in agreement with previous reports that showed that genetically closely related *S*. Enteritidis strains nevertheless presented important metabolic differences, and that these differences were related to the accumulation of single nucleotide polymorphism rather than with differences in gene content [24]. Of note, none of the genes predicted as variant among *S*. Enteritidis in our work correspond to those described as involved in the ability to survive in the avian reproductive tract [50] or in persistence in egg albumen [51]. Furthermore, the genetic regions related to metabolic functions found as variable in our CGH analysis do not correspond to utilization of the compounds described by Morales et al. in their comparative phenotypic analysis of *S*. Enteritidis strains [24].

A report has recently been published showing differences in genetic content among *S*. Enteritidis isolates from prevalent phage types and the non-prevalent phage type 11 [26]. With the exception of the plasmid-encoded genes, all other genes reported as exclusively present in the prevalent phage types, are also present in all the isolates analyzed here.

Overall, our study shows that the epidemic of *S*. Enteritidis in Uruguay between 1995 and 2004 was caused by highly related *S*. Enteritidis isolates, perhaps comprising a PT4-like clonal population with few whole gene differences. To understand more clearly the link between genotype and phenotype and to differentiate between neutral variation within a population and variations associated directly with defined phenotypes, the whole genome sequences of a large number of isolates are required for association studies. This is our future direction.

## Methods

### Bacterial isolates

A sample set of 266 isolates of *S*. Enteritidis isolated in Uruguay was defined among strains received at the National *Salmonella *Centre (Instituto de Higiene, Universidad de la República, Uruguay). Most (218) were isolated during the 9 years from 1995 to 2003 during which there was a nationwide epidemic of food poisoning caused by *S*. Enteritidis. These included a selection of 112 isolates from human cases of gastroenteritis (around 15% of all isolates from faecal culture during the epidemic), all recorded isolates from human systemic infection (48 strains) and all isolates from non-human origin (58 strains). The sample set was completed with all isolates available (6 strains) from prior to the beginning of the epidemic, and 42 isolated after the epidemic declined. The description and source of all Uruguayan strains included in this study are shown in Tables 1 and 2.

A UK isolate that had been completely sequenced and annotated (*S*. Enteritidis PT4 P12519, NCTC 13349) was used as the reference in all analyses [27]. *S*. Enteritidis PT4 P125109 is a human food-poisoning isolate which is highly virulent in newly-hatched chickens.

Six *S*. Enteritidis isolates from other countries were included in CGH analysis. Four clinical isolates (AF3172, AF3173, AF3176, AF3353) were obtained from Centre for Microbiology Research, Kenya Medical Reserch Institute, Nairobi, Kenya. Two veterinary isolates (S1400/94 [52] and 9296/98) were obtained from Veterinary Laboratory Agency, UK. AF3172, AF3173, S1400/94 belong to phage-type 4, AF3176 to phage-type 21, 9296/98 to phage-type 1-c and AF3353 has not been phage-typed.

Isolates were maintained frozen at -80°C in LB containing 25% glycerol. Cultures were performed in LB broth, or on LB containing 1.6% agar, or Tryptic Soy Agar.

All isolates were identified as *Salmonella enterica *using standard biochemical microbiological methods. Serovar was determined by slide agglutination test for O antigens and tube agglutination test for H antigens using commercially available anti O and anti H serum (Difco, France).

Phage typing of the Uruguayan strains was kindly performed by Muna Anjum and collaborators from the Department of Food and Environmental Safety, Veterinary Laboratories Agency, Addlestone, UK.

### Genotyping analysis

All 266 *S*. Enteritidis were subjected to random amplified polymorphism DNA-PCR (RAPD-PCR) analysis using 5 different primers and *S*. Enteritidis PT4 P125109 [27] as reference. A selection of 37 isolates was further subjected to pulse field gel electrophoresis (PFGE) after *Xba*I restriction.

RAPD-PCR was performed as previously described [12]. PFGE of total DNA was performed at the Instituto Carlos Malbran, Buenos Aires, Argentina, following the protocol recommended by PulseNet http://www.cdc.gov/pulsenet/protocols.htm and using a CHEF-DRIII SYS220/240 (BioRad). The electrophoresis profile of each strain was compared to that of PT4 P125109 using Bionumerics software (Applied Maths, St. Martens-Latern, Belgium) and similarity compared using Dice's coefficient. Results are expressed as percentage of identity related to PT4 P125109: 96% of identity corresponds to 1 band of difference, 92% to 2 bands and 91% to 3 bands of difference.

Plasmid DNA was extracted and analyzed by a procedure modified from the method of Kado and Liu [53]. Briefly, 1.5 ml of an LB overnight culture were harvested by centrifugation and suspended in 200 μl E buffer (40 mM Tris, 1 mM EDTA, pH 8,0), mixed gently with 400 μl of lysis solution (50 mM Tris, 100 mM SDS, pH 12,6) and incubated at 58°C for 60 min. 600 μl of phenol/chloroform/isoamyl alcohol (25: 24: 1) solution was mixed gently and the aqueous phase was subjected to phenol/chloroform extraction followed by centrifugation.

### Caco-2 invasion assays

The human colon carcinoma (Caco-2) cell line was obtained from the American Type Culture Collection (ATCC). Caco-2 cells were maintained in DMEM (high glucose, 4500 mg/l), supplemented with 4 mM L-glutamine and 10% foetal calf serum at 37°C in an atmosphere including 5% CO_2_, up to 80% confluence.

For invasion assays, cells were seeded on 24-well plates at a density of 5 × 10^4 ^cells per well, and grown for three days (changing media every other day). The day before the assay, a single colony of each bacterial strain was inoculated in 3 ml of LB broth and grown overnight in an orbital shaking incubator at 37°C (200 rpm). The following day, bacterial cultures were diluted 1/100 in fresh LB and grown with shaking for approximately 2 h to an OD_600 _of 0.4-0.6. Appropriate volumes of bacterial cultures (to give a multiplicity of infection of about 30 bacteria/cell) were spun for 2 minutes at 5500 g, then bacteria were re-suspended by pipetting in Caco-2 growth media and 0.5 ml of this were used to overlay the Caco-2 monolayer. After 1 hour of incubation to allow invasion, the monolayer was washed twice with 1 ml of pre-warmed Dulbecco's PBS (Sigma) and extracellular bacteria were killed by adding medium containing 100 ug/ml of gentamicin (Sigma). After incubation for 90 min, 20 ul of culture supernatants were plated in triplicate in LB agar plates to verify that no viable bacteria were remaining. Cells were washed three times in PBS and then lysed with 0.5 ml of 0.1% Triton X-100 (in water), by incubating for 20 min at 37°C and vigorously pipetting to release intracellular bacteria. Serial 10-fold dilutions of lysates, as well as the corresponding inocula, were plated on LB agar plates for counting viable colonies. For each isolate the percentage of bacteria recovered from intracellular environment to the original inocula was calculated, and this value was normalized so that the invasiveness of the reference strain *S*. Enteritidis PT4 P125109 was 100%. Each strain was tested in duplicate or triplicate, in at least two separate experiments. The mean of all experiments and replicates for each strain was used to assign an invasiveness level expressed as - (≤ 30% of the reference) or + (> 30%). Susceptibility of the isolates to gentamicin was verified using Kirby-Bauer disk diffusion method (NCCLS 2005), and all isolates were susceptible. For statistical analysis to compare the invasiveness of isolates, we used one way ANOVA and Dunnett's multiple comparison test using an alpha = 0,01 (GraphPad Prism software). Fisher's exact test was used to compare the behaviour of isolates obtained from gastroenteritis and invasive disease.

### Comparative Genomic Hybridization analysis

Twenty nine Uruguayan, 4 *S*. Enteritidis isolates from Kenya and 2 from the UK (see Table 2), were analysed by CGH using either the *Salmonella *generation III or IV microarray and *S*. Enteritidis PT4 P125109 as reference [27]. Both *Salmonella *Microarray Generation III and IV http://www.sanger.ac.uk/Projects/Salmonella/ are an extension of the previously described *Salmonella *Generation I Microarray constructed at the Wellcome Trust Sanger Institute [20,22]. These are non-redundant arrays containing coding sequences from the following genomes: *S*. Typhi CT18, *S*. Typhi Ty2, *S*. Typhimurium LT2 (ATCC 700220), *S*. Typhimurium DT104 (NCTC 13348), *S*. Typhimurium SL1344 (NCTC 13347), *S*. Enteritidis PT4 (NCTC 13349), *S*. Gallinarum 287/91 (NCTC 13346) and *S. bongori *12419 (ATCC 43975). The arrays differed on spot layout and positive controls, which were however, not taken into account for analysis purposes.

Total DNA from each strain (including plasmid DNA) was extracted using a Genome DNA extraction kit (Promega) and quantified by agarose gel electrophoresis. Each DNA sample was diluted to 0.1 μg/ml, sonicated for 10 seconds (level 2; Virsonic 300 sonicator) and then labelled with Cy5 (test) or Cy3 (control) using the Bioprime kit (Gibco-BRL) as per manufacturer's instructions. Labeled DNA from *S*. Enteritidis PT4 P125109 (control sample) and one of the query *Salmonella *isolates (experimental sample) were mixed in equal volumes and concentrations. Dye-swap labelling experiments were also performed for each test sample. Mixed labelled DNA was cleaned using an Autoseq G-50 column (Amersham), denatured, and precipitated, and the resulting probes were hybridized to the microarray slide for 17 h at 49°C in a hybridization chamber (Genetix X2530). Washing procedures were stringent with 2 washes at 65°C in 2 × SSC, 0.1% SDS for 30 min and 2 washes at 65°C in 0.1 × SSC for 30 min (1 × SSC is 0.15 M NaCl plus 0.015 M sodium citrate).

Hybridization to microarray slides was detected using a Genepix 4000B scanner (Axon Instruments, Inc.) and quantified using Genepix Pro software (Axon Instruments, Inc.). Signal intensities were corrected by subtracting local background values. Normalization was performed across all features on the array before any filtering took place. Data were normalized to the median value and the total list of 6871 genes was filtered by removing those spots with a high background and genes without data in at least one of the replicates (3 slides per strain, duplicate features per slide). After filtering, a list of 5863 genes was obtained that corresponded to genes that presented a valid signal in at least one of the strains analyzed. Normalization and filtering were performed using GeneSpring microarray analysis software V7.2 (Silicon Genetics). Data analysis was performed on Excel files, following criteria previously described [21] with some modifications, as described below.

Calling of genes present in the PT4 P125109 genome (3978 genes): spots showing low signal when hybridized with PT4 P125109 DNA (median contribution of the reference signal replicates to the total signal among the lowest 5% of all PT4 genes) were assigned as "uncertain". For all other genes, the median of the query strain/PT4 ratios was registered and values higher than 0.67 were assigned as "present" in the query strain whereas those with a ratio value lower than 0.33 were assigned as "absent/divergent" in the query strain. Intermediate ratio values were registered as "uncertain".

Calling of genes absent in the PT4 P125109 genome (1885 genes): if the median contribution of all spots per gene was among the top 70% of all genes represented on the array and the ratio of query strain/PT4 signals was higher than 2.5 the gene was defined as "present" in the query strain. If the median contribution was among the bottom 20% of all genes in the array, the gene was called "absent". Spots that fell outside of these categories were called "uncertain".

For validation, we applied this method to predict genes as being present or absent in the *S*. Typhi CT18 and *S*. Typhimurium DT104 sequenced strains and found an error of less than 1% for prediction of absent/divergent genes, and an error less than 0,1% for prediction of present genes. These mean that from one hundred of genes predicted as absent/divergent in test strain, one can be wrongly included in this category and that from one thousand of genes predicted as present in test strain, one can be wrongly assigned to this category.

Raw microarray data and grid files were submitted to ArrayExpress with accession number E-TABM-603 http://www.ebi.ac.uk/microarray-as/ae/browse.html?keywords=E-TABM-603

### Validation of CGH data by PCR

All PCR reactions were performed using colony-extracted total DNA as template and *invA *as positive control in a multiplex PCR. Primers used to test the presence of ϕSE20 were previously described by Morales et al [24]. Primers used to amplify *gogB *were: gogB-F 5'CTGCAATCTGCCTGCACATATAG-3' and gogB-R 5'CCCAGACCGCATCTGTTAATG-3'. *invA *primers (inv139 and inv141) were previously described by Malorny et al [54]. PCRs were performed in 25 μl reactions with a final concentration of 2 mM MgCl_2_, 200 μM each dNTP, 0.0375 U/μl of *Taq *DNA polymerase (Fermentas), in a Corbett Palm-Cycler. Primers concentrations were: 0.15 μM for *sb9, sb41 *or *gogB *and 0.5 μM for *invA*. The cycling program was as follows: 5 min at 95°C followed by 30 cycles of 30 s at 94°C, 30 s at 60°C and 30 s at 72°C, and completed by a final extension for 5 min at 72°C. Presence and sizes of PCR amplicons were verified by electrophoresis on 2.5% agarose gels in 0.5× TBE.

## Abbreviations

CGH: DNA microarray-based comparative genomic hybridization; SVP: *Salmonella *virulence plasmid; RODs: regions of difference (as defined in ref [24]); PT: phage type; PT4 P125109: *S. enterica *serovar Enteritidis phage type 4 Strain P125109 (NCTC 13349); CT18: *S. enterica *serovar Typhi strain CT18; TY2: *S. enterica *serovar Typhi strain Ty2; LT2: *S. enterica *serovar Typhimurium strain LT2 (ATCC 700220); DT104: *S. enterica *serovar Typhimurium strain DT104 (NCTC 13348); SL1344: *S. enterica *serovar Typhimurium strain SL1344 (NCTC13347); SBG: *S. bongori *strain 12419 (ATCC 43975); SPA: *S. enterica *serovar Paratyphi A strain AKU_12601; SGAL: *S. enterica *serovar Gallinarum strain 287/91; RAPD-PCR: random amplified polymorphism DNA-PCR; PFGE: pulsed field gel electrophoresis; MLST: multi locus sequence typing; MOI: multiplicity of infection; TBE: tris borate EDTA buffer.

## Authors' contributions

LB designed the study, participated in all experiments, performed the analysis of CGH data, interpreted the results and wrote the manuscript. LY carried out the Caco-2 invasion assays, plasmid extraction and participated in the analysis of data, the interpretation of results and the writing of the manuscript. MF carried out the CGH assays, and participated in the analysis of CGH data and in the correction of the manuscript. AM performed the PFGE and RAPD experiments and participated in the analysis of data. NRT participated in the design of the study, collaborated in the interpretation of data and in the writing of the manuscript. AI participated in the design of the study and in the supervision of the analysis of CGH data. SP, CB, GA and FS participated in the design of the study, the supervision of assays, and the writing of the manuscript. DM, SK and GD participated in the design of the study, the interpretation of results and the writing of the manuscript. JAC designed the study, supervised LB, LY and AM, participated in the analysis of data and interpretation of results and wrote the manuscript. All authors have read and approved the final manuscript.
